# Depression education *fotonovela* for engagement of Hispanic patients in treatment: a randomized clinical trial

**DOI:** 10.1186/s12888-021-03641-0

**Published:** 2021-12-23

**Authors:** Katherine Sanchez, Brittany H. Eghaneyan, Michael O. Killian, Leopoldo J. Cabassa, Madhukar H. Trivedi

**Affiliations:** 1 School of Social Work, University of Texas at Arlington, 211 South Cooper Street, Arlington, Texas 76019 USA; 2 Department of Psychiatry, UT Southwestern Medical Center, 6363 Forest Park Rd, Dallas, TX 75235 USA; 3 Department of Social Work, California State University, Fullerton, USA; 4 College of Social Work, Florida State University, 296 Champions Way, UCC 2500, Tallahassee, FL 32306 USA; 5 George Warren Brown School of Social Work, Washington University in St. Louis, Goldfarb Hall, Room 358, Campus Box 1196, One Brookings Drive, St. Louis, MO 63130 USA

**Keywords:** Depression, Education, Hispanics, Stigma, Integrated care, *Fotonovela*

## Abstract

**Background:**

We report the primary outcomes from a randomized clinical trial testing a novel culturally-adapted patient education intervention to increase engagement of Hispanic patients in depression treatment. The Depression Education Fotonovela (DEF), *Secret Feelings*, incorporates popular images, cultural norms, and vivid pictures embedded within a soap opera narrative to increase depression knowledge and dispel myths about treatment. We then assessed engagement in a integrated care treatment model in response to the education intervention and subsequent changes in depression symptoms in a large community-based clinic whose patient population is majority Hispanic.

**Method:**

The sample included 150 adult Hispanic patients with a confirmed diagnosis of depression who were randomly assigned to either: [1] integrated care + fotonovela; or [2] integrated care + standard education. Differences between treatment groups were examined as were changes in depression, anxiety, depression knowledge, and stigma scores over time and engagement in treatment.

**Results:**

Results indicated that while depression scores significantly decreased over time for participants (F [2.811, 416.054] = 197.69, *p* < .001, η^2^ = .572), no differences between the two education groups were found (F [1, 148] = 0.70, *p* = .403, η^2^ = .005). At 12-month follow-up, 101 patients (80.8%) reported a 50% of greater reduction in depression scores from baseline.

**Conclusions:**

We found little difference between the two education groups, suggesting that either may helpful for engaging Hispanic patients into care. Better tailoring of patient education, with the fotonovela or similarly adapted tools, will require more directly addressing the stigma associated with antidepressant medication.

**Trial registration:**

The study was registered with www.clinicaltrials.gov: NCT02702596, on 03/20/2016. Retrospectively registered.

## Background

The prevalence of depression among the US Hispanic population is estimated to be 27% [1]. Response to treatment is slow, and relapse rates are high [2, 3]. Hispanic patients often voice fears about the addictive and harmful properties of antidepressants, worries about taking too many pills, and the stigma attached to taking medications, which may explain the early discontinuation of medication without consulting their provider [4, 5]. Patient education has been associated with better management of chronic disease, increased patient engagement, and improved health outcomes [6], however, less is known about education to proactively address barriers to depression treatment for Hispanic patients [7].

In Latin America, the *fotonovela* is a popular comic-book style pamphlet that portrays a dramatic story using photographs and dialogue bubbles and has become an effective tool for increasing knowledge about public health issues [8, 9]. The Depression Education Fotonovela (DEF), *Secret Feelings*, developed by Cabassa, Molina and Baron [10] differs from typical patient education materials by incorporating popular images, cultural norms and vivid pictures embedded within a soap opera narrative to increase depression knowledge and dispel myths about treatment [8, 11]. *Secret Feelings* has demonstrated significant improvements in depression knowledge and reductions in stigma toward antidepressants and treatment in a community education setting [12], and preliminary success in a pilot study in a community-based, primary care setting [13].

In the current study we report the primary outcomes from a randomized clinical trial aimed to test a novel culturally-adapted patient education intervention to increase engagement of Hispanics in depression treatment. Specifically, we hypothesized the *fotonovela* would increase knowledge of depression, decrease stigma, and increase engagement in treatment better than standard education (SE) among Hispanic primary care patients within an integrated care setting. Secondarily, we assessed changes in symptoms of depression and anxiety over time as a result of receiving the education intervention and subsequent treatment in an integrated care setting.

## Methods

### Study design and setting

METRIC was a randomized controlled trial that took place in a Federally Qualified Health Center (FQHC) in a large metropolitan area in Texas (www.clinicaltrials.gov: NCT02702596). The FQHC operates three locations that provide a full range of comprehensive primary and preventive services to a low-income, Hispanic population. A detailed description of the study setting and methods has been previously reported [14]. The study was reviewed and approved by the Institutional Review Board of the University of Texas at Arlington.

### Recruitment and procedures

**Fig. 1:**
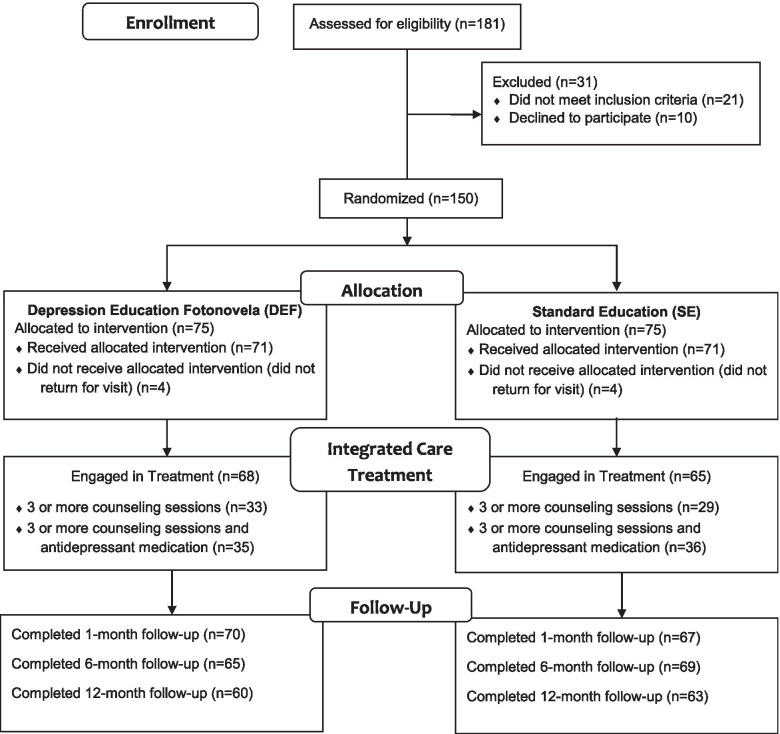
METRIC study flow diagram

Study recruitment took place between February 2016 and February 2018. All adult primary care patients were universally screened for depression using the Patient Health Questionnaire-9 (PHQ-9) [15] as part of normal clinical practice. Patients who screened positive for depression (score greater than or equal to 5) were referred to the Licensed Clinical Social Worker (LCSW) and invited to participate in the one-year study if they met inclusion criteria: confirmed diagnosis of depression, self-identified as Hispanic, and not currently receiving treatment for depression. The study adheres to CONSORT guidelines, see Fig. 1 for the study flow diagram. During the recruitment period, 181 patients were referred for possible enrollment. Of those, 21 did not meet eligibility criteria and 10 declined enrollment, leaving a final sample of 150 participants. After agreeing to participate in the study, participants signed an informed consent document and completed the remaining baseline measures in English or Spanish depending on patient preference.

After collection of baseline measures, participants were randomly assigned to one of two treatment groups: (1) DEF + integrated care; or (2) SE + integrated care. Within 1 week of their enrollment, participants returned to the clinic to complete their educational intervention session (either DEF or SE) and study measures. Bilingual social work students were trained as research assistants (RA) to deliver one of the educational interventions. Research follow up visits were conducted at the following time points: 1 month post education visit, 6 months post enrollment, and 12 months post enrollment. Participants were compensated with a Walmart gift card after the completion of study measures at each visit.

### Intervention

#### DEF

Participants randomized to the DEF + integrated care treatment group were given a copy of the *Secret Feelings fotonovela* [10] which is written at a 4th grade reading level in both English and Spanish in a colorful, comic-book style pamphlet. *Secret Feelings* presents information on depression symptoms and treatment while portraying a dramatic, soap opera-style story. During the education visit, the RA read the *fotonovela* with the participant, answered any questions, and invited the participant to share th pamphlet with others. The DEF session lasted about 30–45 min.

#### SE

Participants randomized to the SE + integrated care treatment group were given a copy and read the patient education materials from the National Institute of Mental Health [16], a colorful, tri-fold brochure that was available in both English and Spanish. The brochure included information on signs and symptoms of depression, causes and types of depression, and treatment options. Participants were given the opportunity to ask questions and take the pamphlet home. The SE session lasted about 20 min.

#### Integrated care

All participants enrolled in the study received integrated care in which the LCSW worked closely with patients and their primary care provider to develop a treatment plan which included counseling and/or pharmacotherapy while closely monitoring depressive symptoms, antidepressant treatment adherence (if applicable), and treatment response.

### Data collection and measures

All study data and measures were collected and stored via REDCap (Research Electronic Data Capture) [17]. Demographic information collected from the participants’ medical record included: age, sex, marital status, and education level.

#### Depression

Depression symptom severity was assessed using the PHQ-9 [15], a self-report measure that was already in use in the clinic as part of routine screening for depression. Among Hispanic samples, the English and Spanish versions of the PHQ-9 have demonstrated strong internal consistency and similar factor structures [18–20].

#### Anxiety

Anxiety severity was assessed using the Generalized Anxiety Disorder 7-item scale (GAD-7) [21]. The GAD-7 has demonstrated strong internal consistency reliability for both the English and Spanish versions among Hispanic Americans [22].

#### Depression knowledge

Knowledge of depression symptoms and treatment was assessed using the the 17-item Depression Knowledge Measure (DKM) developed by Unger et al. [12]. The first 10 items assess depression symptom recognition. The second half of the measure presents seven true-false questions to assess treatment knowledge. One point is allocated for reach correct response, with total scores ranging from 0 (all incorrect) to 17 (all correct).

#### Stigma

Three measures developed by Interian et al. [23] were used to assess stigma towards depression treatment: Stigma Concerns about Mental Health Care (SCMHC), Social Distance Scale (SDS), and the Latino Scale for Antidepressant Stigma (LSAS) [24]. Previous psychometric research has shown support for the use of the measures among Spanish-speaking primary care patients in measuing unique constructs of stigma toward mental health and treatment [23]. The SCMHC is a 3-item scale that assesses an individual’s anticipated stigma if they were to seek treatment for depression [23]. Possible scores on the measure range from 0 to 3, with higher scores indicating greater stigma. The Social Distance Scale (SDS) is a 6-item scale that measures social distance desirability from someone with a history of depression treatment. Total scores range from 0 to 12, with lower scores indicating greater desired social distance (i.e., greater stigma). Finally, the LSAS is a 7-item scale that assesses perceived stigma towards the use of antidepressant medication. Possible scores for the scale range from 0 to 14, with higher scores indicating greater stigma [23].

#### Treatment engagement

Participants’ engagement in treatment was determined after the 12-month intervention time period and was categorized into three groups: those who did not engage in treatment, those who engaged in counseling only (attended 3 or more counseling visits), and those who engaged in counseling and antidepressant medication (attended 3 or more counseling visits and took antidepressants for at least 2 months).

### Statistical analyses

Bivariate statistical analyses were used to examine both differences between intervention groups (i.e., SE and DEF) and those completing treatment compared to those dropping out of the study before completing the 12-month follow-up. These tests included *t*-tests and 𝜒2 analyses, depending on the measure with effect size metrics of Cohen’s *d* or Cramer’s *V*, respectively. An intention-to-treat approach using a last-observation-carried-forward method was used to analyze outcome data whereby the last available measurement for all participants was then used for all time points through the 12-month final follow-up. Missing data was handled using this carry-forward method. Repeated-measures ANOVA model was used to examine both within-subjects (i.e., over time) and between-subjects (i.e., between intervention groups) differences in depression, anxiety, depression knowledge, and stigma scores over four or five time points, depending on the measure. Partial eta-squared (η^2^) scores were used as metrics of effect size.

The study was adequately powered. A total of 150 participants were randomly assigned to the treatment and control groups. We estimated that 60 participants were required in each of the intervention and control groups with five measurement points to have an 80% chance, with a 5% significance level, of detecting an effect size of *d* = .258 between the two groups, a lower effect than reported in other studies of the fotonovela [25]. This assumed sample size was greater than the obtained sample at 12-month follow-up. Assuming an intent-to-treat analysis with 150 participants, the same assumptions would leave the minimal detectable effect size at *d* = .230.

## Results

### Sample characteristics

At baseline, 98.0% (*n* = 148 of 150) of the sample reported moderate to severe depressive symptoms (Table 1). Severe depression scores were reported by 9.3% of the sample (*n* = 29). The sample was entirely Hispanic and their ccountry of origin unknown, though 88% of Hispanics in Texas are of Mexican descent [26]. The vast majority were women (*n* = 133, 88.7%), Spanish speaking (*n* = 136, 90.7%), and currently married (*n* = 106, 72.1%). A majority of the sample reported some high school or less (*n* = 79, 52.7%). The mean age was 39.36 ± 9.08 years at time of baseline measurement.

**Table 1:** Sample Characteristics and Randomization Check

Demographic and Patient Characteristic	Total Sample (*n* = 150)	Standard Education (*n* = 75)	Fotonovela (*n* = 75)	Test	Effect size
Age, *M* ± *SD*	39.36 ± 9.08	40.52 ± 8.46	38.20 ± 9.57	t = 1.57	*d* = 0.26
Sex, female, *n (%)*	133 (88.7%)	63 (84.0%)	70 (93.3%)	𝜒^2^ = 3.25	V = 0.15
Spanish Speaking, yes, *n (%)*	136 (90.7%)	69 (92.0%)	67 (89.3%)	𝜒^2^ = 0.32	V = 0.05
Marital Status, *n (%)*				𝜒^2^ = 0.46	V = 0.06
*Married/cohabitating*	106 (72.1%)	54 (74.0%)	52 (70.3%)		
*Never married*	12 (8.2%)	6 (8.2%)	6 (8.2%)		
*Widowed*	0 (0.0%)	0 (0.0%)	0 (0.0%)		
*Divorced*	21 (14.3%)	9 (12.3%)	12 (16.2%)		
*Other*	8 (5.3%)	4 (5.5%)	4 (5.5%)		
Education Level, *n (%)*				𝜒^2^ = 11.97^+^	V = 0.29
*5th grade or less*	21 (14.3%)	13 (17.6%)	8 (11.0%)		
*6th to 8th grade*	35 (23.8%)	20 (27.0%)	15 (20.5%)		
*Some high school*	23 (15.6%)	13 (17.6%)	10 (10.7%)		
*High school or GED*	52 (35.4%)	25 (33.8%)	27 (37.0%)		
*Vocational or trade school*	1 (0.7%)	1 (1.4%)	0 (0.0%)		
*Some college*	10 (6.8%)	2 (2.7%)	8 (11.0%)		
*College degree*	5 (3.4%)	0 (0.0%)	5 (6.8%)		
Attrition, yes, *n (%)*	25 (16.7%)	11 (14.7%)	14 (18.7%)	𝜒^2^ = 0.43	V = 0.05
Sessions attended, *M* ± *SD*	11.90 ± 6.97	11.20 ± 7.28	12.60 ± 6.23	t = 1.23	*d* = 0.21
PHQ-9, baseline, *M* ± *SD*	15.32 ± 4.15	15.36 ± 4.09	15.28 ± 4.24		
PHQ-9 severity category, *n (%)*				𝜒^2^ = 3.79	V = 0.16
*Mild depression, n (%)*	3 (2.0%)	2 (2.7%)	1 (1.3%)		
*Moderate depression, n (%)*	70 (46.7%)	32 (42.7%)	38 (50.7%)		
*Moderately severe depression, n (%)*	48 (32.0%)	29 (38.7%)	19 (25.3%)		
*Severe depression, n (%)*	29 (19.3%)	12 (16.0%)	17 (22.7%)		
GAD7 scores, *M* ± *SD*	12.52 ± 4.56	12.17 ± 4.57	12.87 ± 4.56	t = 0.93	*d* = 0.15
DKM scores, *M* ± *SD*	11.73 ± 1.96	11.33 ± 2.12	12.12 ± 1.71	t = 2.50*	*d* = 0.41
SCHMC scores, *M* ± *SD*	0.43 ± 0.83	0.38 ± 0.81	0.48 ± 0.86	t = .70	*d* = 0.06
LSAS scores, *M* ± *SD*	6.07 ± 3.18	5.57 ± 3.06	6.56 ± 3.24	t = 1.90	*d* = 0.31
SDS scores, *M* ± *SD*	13.02 ± 3.51	12.95 ± 3.60	13.09 ± 3.44	t = 0.26	*d* = 0.04

*Note. PHQ-9* Patient Health Questionnaire-9, *GAD7* Generalized Anxiety Disorder 7-item scale, *DKM* Depression Knowledge Measure, *SCHMC* Stigma Concerns about Mental Health Care scale, *LSAS* Latino Scale for Antidepressant Stigma, *SDS* Social Distance Scale

* *p* < .05

### Randomization and attrition

Patients were evenly randomized to each of the SE group (*n* = 75) and the DEF group (*n* = 75). Eight participants (5.3% of 150) dropped from the study prior to receipt of either SE or the DEF intervention, four from each group. Tests between the two groups (*n* = 150) on patient indicated randomization produced largely comparable groups on relevant patient characteristics and study measures (Table 1). A significant difference between the two intervention groups was found on the DKM measure (t = 2.50, df = 148, *p* = .013, Cohen’s d = 0.41) where the DEF group (12.12 ± 1.71) demonstrated significantly more depression knowledge than the SE group at baseline (11.33 ± 2.12). The DEF group (6.56 ± 3.24) demonstrated more bias towards psychiatric medications compared to SE group at baseline (5.57 ± 3.06), but this difference was not significant (t = 1.90, df = 146, *p* = .060) despite the moderate effect size (Cohen’s d = 0.42).

Analyses were completed to identify differences between those completing the intervention and 12-month follow-up (*n* = 123) and those who were lost to follow-up (*n* = 27). Those who did not complete the study significantly differed by proportion of sex in each group (𝜒^2^ = 4.21, df = 1, *p* = .040, *V* = .17). All of those attritioning from the study were women. Moderate effect sizes were present for the difference between those completing the protocol and dropping out on GAD-7 scores with attritioners reporting greater levels of anxiety (14.33 ± 4.53) compared to those completing the study (12.12 ± 4.49). There were no significant differences between completers and attritioners in any other baseline characteristics or by intervention group (*p* > .05).

### Depression knowledge and stigma outcomes

Depression knowledge scores (Table 2) reported on the DKM assessment significantly differed over time (F [3.52, 416.054] = 10.66, *p* < .001, partial η^2^ = .080) and by intervention group (F [1, 147] = 13.09, *p* < .001, partial η^2^ = .082). Across all timepoints, including baseline, the group receiving the DEF was found to have significantly higher depression knowledge (Cohen’s *d* = 0.375 to 0.618) with the greatest difference observed directly after the educational visit. Despite the differences, no time by group interaction was found (F [3.52, 517.99] = 0.66, *p* = .598, partial η^2^ = .004). Due to baseline imbalance of DKM scores between groups, a sensitivity analysis was conducted controlling for baseline DKM scores across subsequent time points. The results indicated no significant group differences at 1-month (F [1147] = 1.05, *p* = .306), 6-month (F[1147] = 1.16, *p* = .206), or 12-month (F[1147] = 1.60, *p* = .207) follow-up scores.

**Table 2:** Outcomes over time using all carry forward intention-to-treat analysis

	Standard Education (*n* = 75)					Fotonovela (*n* = 75)				
Variable	Baseline	EV	1 m	6 m	12 m	Baseline	EV	1 m	6 m	12 m
PHQ9	15.36 ± 4.09	–	8.33 ± 5.37	4.84 ± 5.21	4.79 ± 5.46	15.28 ± 4.24	–	8.64 ± 5.99	5.68 ± 5.40	5.85 ± 6.15
*Model*	Within-subjects F = 197.69***; Between-groups F = 0.703; η^2^ = 0.005									
GAD7	12.17 ± 4.57	–	7.13 ± 5.25	4.49 ± 4.85	4.08 ± 4.61	12.87 ± 4.56	–	7.79 ± 5.60	5.73 ± 5.26	5.71 ± 5.50
*Model*	Within-subjects F = 131.66***; Between-groups F = 2.833; η^2^ = 0.019									
DKM	11.42 ± 2.00	12.08 ± 1.86	11.88 ± 1.91	11.97 ± 2.21	12.16 ± 2.15	12.12 ± 1.71	13.23 ± 1.94	12.95 ± 1.95	12.87 ± 1.94	13.03 ± 1.97
*Model*	Within-subjects F = 10.66***; Between-groups F = 12.09***; η^2^ = 0.082									
SCMHC	0.38 ± 0.81	0.44 ± 0.88	0.37 ± 0.86	0.26 ± 0.67	0.16 ± 0.55	0.48 ± 0.86	0.16 ± 0.47	0.15 ± 0.56	0.13 ± 0.55	0.11 ± 0.48
*Model*	Within-subjects F = 6.26***; Between-groups F = 2.210; η^2^ = 0.015									
LSAS	5.58 ± 3.06	5.71 ± 2.73	5.77 ± 2.74	5.88 ± 2.90	5.44 ± 2.70	6.56 ± 3.24	6.49 ± 3.16	6.57 ± 3.06	6.27 ± 3.06	6.13 ± 3.13
*Model*	Within-subjects F = 0.91; Between-groups F = 3.48^+^; η^2^ = 0.023									
SD	13.21 ± 3.88	10.61 ± 1.92	10.76 ± 1.91	10.69 ± 2.21	10.86 ± 1.88	13.00 ± 3.60	10.66 ± 2.37	10.73 ± 2.09	10.72 ± 2.26	10.96 ± 2.09
*Model*	Within-subjects F = 35.11***; Between-groups F = 0.05; η^2^ = 0.001									

*Note. PHQ-9* Patient Health Questionnaire-9, *GAD7* Generalized Anxiety Disorder 7-item scale, *DKM* Depression Knowledge Measure, *SCHMC* Stigma Concerns about Mental Health Care scale, *LSAS* Latino Scale for Antidepressant Stigma, *SDS* Social Distance Scale

*** *p* < .001

Differences by intervention group were not significant for the SCMHC (F [1, 146] = 2.21, *p* = .140, partial η^2^ = .015) or the SDS measures (F [1, 146] = 0.053, *p* = .819, partial η^2^ = .001). However, scores for the SDS (F [4, 584] = 35.11, *p* < .001, partial η2 = .194) and SCMHC (F [4, 584] = 6.26, *p* < .001, partial η2 = .041) significantly decreased for both groups over time. While decreases in SCMHC score represent a decrease in participant stigma towards mental health care, decreases in SDS scores indicate greater stigma towards others with depression or receiving depression treatment.

In contrast, attitudes towards psychiatric medications did not significantly change over time (F [3.48, 507.31] = 0.987, *p* = .449, partial η^2^ = .007), but the DEF group reported nearly significantly greater stigma towards medication than the SE group (F [1, 146] = 3.48, *p* = .064, partial η^2^ = .023). The effect size of the difference was small, however.

### Treatment engagement

One hundred and thirty-three participants (93.7%) engaged in treatment: 62 participants (43.7%) received counseling only without antidepressant medication and 71 participants (50.0%) received counseling and antidepressant medications. The type of educational intervention received by patients was not significantly associated with treatment engagement (𝜒^2^ = 0.21, df = 1, *p* = .65, *V* = .004).

### Depression and anxiety outcomes

Results indicated that while PHQ-9 scores (Table 2) significantly decreased over time for participants (F [2.811, 416.054] = 197.69, *p* < .001, partial η^2^ = .572), no differences between the SE and DEF groups were found (F [1, 148] = 0.70, *p* = .403, partial η^2^ = .005). For all participants at the 12-month follow-up (*n* = 123), 99 patients (80.5%) reported a 50% or greater reduction in depression scores from baseline, yet this reduction was not significantly associated with the educational intervention (𝜒2 = 2.52, df = 1, *p* = .112, *V* = .143) nor was it associated with type of treatment engagement (𝜒2 = 3.26, df = 1, *p* = .071, *V* = .157). Similarly, reported anxiety scores decreased over time (F [2.847, 421.286] = 131.66, p < .001, partial η^2^ = .471) and no differences by educational intervention group (F [1, 148] = 2.83, *p* = .094, partial η^2^ = .019).

Results indicated that while PHQ-9 scores (Table 2) significantly decreased over time for participants (F [2.811, 416.054] = 197.69, *p* < .001, partial η^2^ = .572), no differences between the SE and DEF groups were found (F [1, 148] = 0.70, *p* = .403, partial η^2^ = .005). For all participants at the 12-month follow-up (*n* = 123), 99 patients (80.5%) reported a 50% or greater reduction in depression scores from baseline, yet this reduction was not significantly associated with the educational intervention (𝜒2 = 2.52, df = 1, *p* = .112, *V* = .143) nor was it associated with type of treatment engagement (𝜒2 = 3.26, df = 1, *p* = .071, *V* = .157). Similarly, reported anxiety scores decreased over time (F [2.847, 421.286] = 131.66, p < .001, partial η^2^ = .471) and no differences by educational intervention group (F [1, 148] = 2.83, *p* = .094, partial η^2^ = .019).

Results indicated that while PHQ-9 scores (Table 2) significantly decreased over time for participants (F [2.811, 416.054] = 197.69, *p* < .001, partial η^2^ = .572), no differences between the SE and DEF groups were found (F [1, 148] = 0.70, *p* = .403, partial η^2^ = .005). For all participants at the 12-month follow-up (*n* = 123), 99 patients (80.5%) reported a 50% or greater reduction in depression scores from baseline, yet this reduction was not significantly associated with the educational intervention (𝜒2 = 2.52, df = 1, *p* = .112, *V* = .143) nor was it associated with type of treatment engagement (𝜒2 = 3.26, df = 1, *p* = .071, *V* = .157). Similarly, reported anxiety scores decreased over time (F [2.847, 421.286] = 131.66, p < .001, partial η^2^ = .471) and no differences by educational intervention group (F [1, 148] = 2.83, *p* = .094, partial η^2^ = .019).

## Discussion

In this trial of a unique, culturally adapted patient education tool designed to increase knowledge of depression, decrease stigma, and increase engagement of Hispanic patients in treatment, we found no difference in patients who received the *fotonovela* intervention compared to patients who received usual patient education, with both groups demonstrating greater knowledge of depression across all timepoints. We also found no differences in engagement in treatment or clinical outcomes between groups after the education intervention. In fact, treatment in an integrated care model led to significant improvement in depression and anxiety symptoms, regardless of education group during a one-year intervention period among a mostly female, Spanish-speaking sample of Hispanic patients in a large community-based clinic.

The characteristics of the sample were essentially uniform across the two education intervention groups, including their severity of depression, however, the *fotonovela* group had greater baseline depression knowledge prior to receiving any education intervention. This imbalance among the groups subsequently held across all timepoints and was greatest immediately after the delivery of the educational intervention. Depression knowledge increased over time in both groups, suggesting a surge in knowledge after education which was sustained regardless of the intervention group .

In the current sample, stigma towards medication did not improve over time and, in fact, was greater among the recipients of the *fotonovela* but, interestingly, did not act as a deterrent to engaging in treatment. Virtually the entire patient sample engaged in treatment of some kind, with more than half receiving a combination of medication and counseling. Increased stigma toward antidepressant use may reflect knowledge gained via the *fotnovela* which reinforced fears about side effects and stigmatizing attitudes toward medication in general, and are similar to findings from our pilot feasability study of the *fotonovela* [13]. While these attitudes are not unique among Hispanic patients receiving treatment who frequently articulate fear of being stigmatized and deep concerns about depression medication being addictive [27], we conclude the *fotonovela*, *Secret Feelings*, likely requires further tailoring to better address known side effects of antidepressants [24, 28, 29].

### Limitations

The current study’s findings are limited by the study design and methodology as well as the relatively small, homogenous sample. It is possible that the robust nature of both education interventions led to no significant differences between groups. However, the structure of a clinical trial required comparison to an alternative treatment and we chose an enhanced treatment as usual condition. As with every randomized clinical trial, the process of randomization may have not produced equivalent groups, as evidenced by the *Fotonovela* group having higher DKM scores on baseline, which may have extended to other participant factors not measured in the current study. Additionally, the LCSW not being blinded to the intervention condition of participants as well as the use of non-blinded assessors may have led to additional threats to internal validity such as measurement bias or diffusion effects. Finally, study participants were predominantly female and Spanish speaking, suggesting results may not be generalizable to male and English-speaking Hispanic populations.

## Conclusions

Contrary to our study hypothesis, we found little difference between the two education conditions, a culturally adapted tool and standardized depression education. Racial and ethnic minorities continue to experience persistent gaps in access to quality depression care, and those disparities in receipt of treatment are on the rise [30]. Since stigma towards medication did not improve over time and, in fact, was greater among the recipients of the *fotonovela,* further tailoring of patient education may require more directly addressing patient level barriers which pose considerable challenges to treatment, often lead to subtherapeutic doses of medication, poor treatment adherence, and quality of life [29].

## Data Availability

The datasets used and/or analysed during the current study are available from the corresponding author on reasonable request.

## Abbreviations

DEF: Depression education fotonovela
DKM: Depression Knowledge Measure
FQHC: Federally qualified health center
GAD-7: Generalized Anxiety Disorder-7
LCSW: Licensed clinical social worker
LSAS: Latino Scale for Antidepressant Stigma
METRIC: Measurement, Education, and Tracking in Integrated Care
NIMH: National Institute of Mental Health
PHQ-9: 9-item Patient Health Questionnaire
PI: Principal Investigator
SCMHC: Stigma Concerns about Mental Health Care
SDS: Social Distance Scale
SE: Standard education
