# Correction: Depression education *fotonovela* for engagement of Hispanic patients in treatment: a randomized clinical trial

**DOI:** 10.1186/s12888-023-04737-5

**Published:** 2023-04-19

**Authors:** Katherine Sanchez, Brittany H. Eghaneyan, Michael O. Killian, Leopoldo J. Cabassa, Madhukar H. Trivedi

**Affiliations:** 1 School of Social Work, University of Texas at Arlington, 211 South Cooper Street, Arlington, TX 76019 USA; 2 Department of Psychiatry, UT Southwestern Medical Center, 6363 Forest Park Rd, Dallas, TX 75235 USA; 3 Department of Social Work, California State University, Fullerton, USA; 4 College of Social Work, Florida State University, 296 Champions Way, UCC 2500, Tallahassee, FL 32306 USA; 5 George Warren Brown School of Social Work, Washington University in St. Louis, Goldfarb Hall, Room 358, Campus Box 1196, One Brookings Drive, St. Louis, MO 63130 USA

**Correction: BMC Psychiatry 21, 635 (2023)**

**https://doi.org/10.1186/s12888-021-03641-0**

Following the publication of the original article [1], an error was identified in the **Depression and anxiety outcomes** heading. The paragraph was mentioned three times and has been corrected since then.

The original article [1] has been corrected.
